# Prediction of long-term recurrent ischemic stroke: the added value of non-contrast CT, CT perfusion, and CT angiography

**DOI:** 10.1007/s00234-020-02526-5

**Published:** 2020-08-28

**Authors:** Frans Kauw, Jacoba P. Greving, Richard A. P. Takx, Hugo W. A. M. de Jong, Wouter J. Schonewille, Jan A. Vos, Marieke J. H. Wermer, Marianne A. A. van Walderveen, L. Jaap Kappelle, Birgitta K. Velthuis, Jan W. Dankbaar, On behalf of the Dutch acute stroke study (DUST) investigators, C. B. Majoie, Y. B. Roos, L. E. Duijm, K. Keizer, A. van der Lugt, D. W. Dippel, K. E. Droogh-de Greve, H. P. Bienfait, G. J. Lycklama à Nijeholt, J. Boiten, D. Duyndam, V. I. Kwa, F. J. Meijer, E. J. van Dijk, F. O. Kesselring, J. Hofmeijer, W. J. van Rooij, P. L. de Kort, C. C. Pleiter, S. L. Bakker, J. Bot, M. C Visser, I. C. van der Schaaf, W. P. Mali, T. van Seeters, A. D. Horsch, J. M. Niesten, G. J. Biessels, M. J. Luitse, Y. van der Graaf

**Affiliations:** 1 Department of Radiology, University Medical Center Utrecht, Utrecht University, Heidelberglaan 100, 3584 CX Utrecht, The Netherlands; 2 Julius Center for Health Sciences and Primary Care, University Medical Center Utrecht, Utrecht University, Utrecht, The Netherlands; 3 Department of Neurology, St. Antonius Hospital, Nieuwegein, The Netherlands; 4 Department of Radiology, St. Antonius Hospital, Nieuwegein, The Netherlands; 5 Department of Neurology, Leiden University Medical Center, Leiden, The Netherlands; 6 Department of Radiology, Leiden University Medical Center, Leiden, The Netherlands; 7 Brain Center, Department of Neurology and Neurosurgery, University Medical Center Utrecht, Utrecht University, Utrecht, The Netherlands

**Keywords:** Brain infarction, Recurrent event, Risk factor, Computed tomography, Survival analysis, Ischemic Stroke, Complications, Risk Factors, Computerized tomography, Prognosis

## Abstract

**Purpose:**

The aim of this study was to evaluate whether the addition of brain CT imaging data to a model incorporating clinical risk factors improves prediction of ischemic stroke recurrence over 5 years of follow-up.

**Methods:**

A total of 638 patients with ischemic stroke from three centers were selected from the Dutch acute stroke study (DUST). CT-derived candidate predictors included findings on non-contrast CT, CT perfusion, and CT angiography. Five-year follow-up data were extracted from medical records. We developed a multivariable Cox regression model containing clinical predictors and an extended model including CT-derived predictors by applying backward elimination. We calculated net reclassification improvement and integrated discrimination improvement indices. Discrimination was evaluated with the optimism-corrected c-statistic and calibration with a calibration plot.

**Results:**

During 5 years of follow-up, 56 patients (9%) had a recurrence. The c-statistic of the clinical model, which contained male sex, history of hyperlipidemia, and history of stroke or transient ischemic attack, was 0.61. Compared with the clinical model, the extended model, which contained previous cerebral infarcts on non-contrast CT and Alberta Stroke Program Early CT score greater than 7 on mean transit time maps derived from CT perfusion, had higher discriminative performance (c-statistic 0.65, *P* = 0.01). Inclusion of these CT variables led to a significant improvement in reclassification measures, by using the net reclassification improvement and integrated discrimination improvement indices.

**Conclusion:**

Data from CT imaging significantly improved the discriminatory performance and reclassification in predicting ischemic stroke recurrence beyond a model incorporating clinical risk factors only.

**Electronic supplementary material:**

The online version of this article (10.1007/s00234-020-02526-5) contains supplementary material, which is available to authorized users.

## Background

Recurrent stroke accounts for approximately a quarter of all strokes that occur and has important implications for the long-term outcome of patients [1]. The 1-year incidence of recurrent ischemic stroke has been estimated to range from 8 to 14% [2, 3]. The risk estimation of recurrent ischemic stroke can be achieved by using prediction models.

Many clinical predictors for recurrent ischemic stroke have been investigated. Strong evidence was established for a limited number of factors, which include stroke prior to the index stroke and stroke subtype [4]. Other factors such as age, sex, hypertension, diabetes mellitus, hyperlipidemia, smoking, history of myocardial infarction, history of atrial fibrillation, and history of peripheral artery disease have been suggested as predictors of recurrent ischemic stroke in some studies, but not in others [4].

CT is often the imaging modality of choice for diagnosing acute ischemic stroke mostly due to high availability and lack of contraindications [5]. Previously identified imaging predictors for recurrent ischemic stroke include acute ischemia on non-contrast CT (NCCT), occlusion or stenosis on CT angiography (CTA), and poor collateral supply on CTA [6, 7]. In addition, magnetic resonance imaging (MRI)–derived predictors such as multiple ischemic lesions are shown to have added value to clinical models [4, 8, 9].

Clinical prediction models have been summarized in a systematic review and meta-analysis [10]. The discriminative performance of these models was moderate [10]. Most models are developed for predicting recurrences of ischemic stroke not longer than 90 days or 1 year after the initial stroke, whereas ischemic stroke may recur up to 5 years after the initial stroke and beyond [11]. To our knowledge, the only model that was developed to predict 5-year ischemic stroke recurrence was developed in young stroke patients. Prediction of 5-year recurrent stroke in adult patients has not been studied before, and the added value of CT-derived predictors is unknown. Therefore, we sought to develop a model incorporating clinical risk factors for predicting 5-year recurrent ischemic stroke in patients with ischemic stroke and to determine whether adding CT-derived predictors improves prediction of recurrent ischemic stroke over 5 years of follow-up.

## Methods

### Study population

All patients participated in the Dutch acute stroke study (DUST), a prospective multicenter observational cohort study in The Netherlands. The DUST was designed to assess the prognostic value of CT perfusion (CTP) and CTA in predicting clinical outcome after 90 days, in addition to patient characteristics and NCCT findings. A detailed description of the design and baseline characteristics of the participants has been described elsewhere [12, 13]. The study was approved by the medical ethics committee of the University Medical Center Utrecht, The Netherlands.

The current study is based on the three largest DUST centers: University Medical Center Utrecht; Leiden University Medical Center; and St. Antonius Hospital, Nieuwegein. Patients were enrolled between 2009 and 2013. Additional 5-year follow-up data were collected by evaluating medical records in June 2018. From the original 766 patients enrolled in the three selected DUST centers, imaging data were incomplete for 38 patients. Additionally, we excluded 90 patients without a diagnosis of ischemic stroke at baseline. The remaining sample included 638 patients.

### Baseline assessments

The following baseline data were collected: demographics (age and sex), pre-stroke modified Rankin scale (mRS), characteristics of the index event (National Institutes of Health stroke scale [NIHSS], Trial of ORG 10172 in Acute Stroke Treatment [TOAST] classification), time from symptom onset to CT scan, intravenous thrombolysis, endovascular treatment, and vascular risk factors [14]. Imaging data were extracted from the DUST database. In the DUST, the acquired images were assessed by an observer with at least 5 years of experience in neurovascular imaging (from a pool of three observers) [12]. The observers were blinded for clinical information (except for side of symptoms), follow-up imaging, and clinical outcomes. Imaging data included NCCT findings such as hyperdense vessel sign and Alberta Stroke Program Early CT score (ASPECTS) of either the anterior or posterior circulation. CTA findings included the presence of occlusion, clot burden score (CBS), collateral score (CS), and internal carotid artery (ICA) stenosis [15–17]. Furthermore, ASPECTS was determined on cerebral blood volume and mean transit time maps, which were derived from CTP [18]. The CTP coverage was adjusted to the clinical indication [12]. The CTP coverage ranged from 40 mm to full brain coverage including at least both ASPECTS levels. In case a posterior circulation stroke was expected, the CTP coverage was adjusted to cover all three pc-ASPECTS levels.

### Candidate predictors

All candidate predictors were selected based on the results of two systematic literature reviews [4, 10]. Demographic factors included age and sex. Lifestyle factors included current smoking. Clinical characteristics included the NIHSS score (continuous) on admission and the TOAST classification. Health variables included hypertension, diabetes mellitus, hyperlipidemia, history of myocardial infarction, history of atrial fibrillation, history of peripheral artery disease, and history of transient ischemic attack (TIA) or ischemic stroke.

Imaging characteristics included previous cerebral infarcts on NCCT, hyperdense vessel sign on NCCT, ICA stenosis > 70% on CTA, collateral score (poor versus good) on CTA, and ASPECTS (> 7 versus ≤ 7) on cerebral blood volume and mean transit time maps [17, 19, 20].

### Recurrent ischemic stroke

Follow-up data were based on hospital visits (e.g., follow-up visits or admissions) or communications (e.g., telephone or correspondence) and were extracted from medical records. If follow-up data were missing, the patient was censored in this study at the time of the last visit or communication.

The primary outcome was recurrent ischemic stroke, which was defined as a clinical event of sudden onset with new neurological deficits that persisted for more than 24 h and was not caused by another diagnosis than ischemic stroke.

### Statistical analysis

Hazard ratios and 95% confidence intervals were calculated for candidate predictors of recurrent ischemic stroke by performing multivariable Cox regression analyses. We plotted Schoenfeld residual plots to check the proportional hazards assumption, which was not violated.

Two models were developed: model 1 included clinical predictors and model 2 included clinical and imaging predictors. First, the clinical model was developed applying backward elimination to the model with all the clinical candidate predictors. The full model containing all candidate predictors was then simplified by performing backward elimination based on a *P* value threshold of 0.1. For model 2, the candidate imaging predictors were added to the final clinical model and the described process of backward elimination was repeated. To prevent removal of the clinical predictors, the clinical predictors were forced into the model. Model improvement was evaluated by calculating the continuous net reclassification improvement (NRI) and 95% confidence interval using the Kaplan–Meier method and 1000 bootstrap samples [21, 22]. NRI is based on reclassification of patients with or without the outcome and increases as the patients with the outcome are reclassified as having a high risk or the patients without the outcome are reclassified as having a low risk [21]. The improvement in discrimination slopes was evaluated with the measure of integrated discrimination improvement (IDI) [21]. Model improvement was also evaluated with the likelihood ratio test.

Optimism of the clinical and extended models was evaluated with 1000 bootstrap samples. Global shrinkage of the model was done with the jackknife method [23]. Finally, discrimination and calibration of the optimism-corrected model were assessed with the c-statistic and the calibration plot and its slope. Statistical analysis was performed with packages *survival*, *rms*, *nricens*, and *survIDINRI* in R version 3.5.0. This study was performed in accordance with the TRIPOD Checklist for Prediction Model Development and Validation [24].

## Results

Of the 638 patients with stroke at baseline, 56 (9%) had a recurrent ischemic stroke over the 5 years of follow-up. Baseline characteristics are shown in Table 1. Comparison of the baseline characteristics of our study population with all remaining DUST participants is shown in Supplemental Table I.

**Table 1:** Baseline characteristics stratified by recurrence of ischemic stroke over 5 years of follow-up

Characteristic	Recurrence (*n* = 56)	No recurrence (*n* = 582)
Age, mean ± SD	68 ± 12	67 ± 14
Male sex, *n* (%)	41 (73)	324 (56)
mRS ≥ 3 before stroke, *n* (%)	1 (2)	43 (7)
Admission NIHSS, median (Q1–Q3)	4 (3–7)	6 (3–12)
Posterior circulation infarct, *n* (%)	11 (20)	79 (14)
Intravenous thrombolysis, *n* (%)	30 (54)	360 (62)
Endovascular treatment, *n* (%)	1 (2)	40 (7)
Medical history		
Hypertension, *n* (%)	34 (62)	285 (49)
Diabetes mellitus, *n* (%)	10 (18)	74 (13)
Hyperlipidemia, *n* (%)	29 (52)	161 (28)
Smoking currently, *n* (%)	15 (29)	178 (33)
Former smoking, *n* (%)	22 (42)	154 (28)
Never smoked, *n* (%)	15 (29)	213 (39)
Atrial fibrillation, *n* (%)	8 (15)	79 (14)
Anticoagulant medication, *n* (%)	12 (21)	82 (14)
History of stroke or TIA, *n* (%)	22 (39)	114 (20)
History of MI, *n* (%)	10 (18)	65 (11)
History of PAD, *n* (%)	4 (7)	30 (5)
Imaging findings		
Previous cerebral infarcts on NCCT, *n* (%)	24 (43)	170 (29)
Hyperdense vessel sign, *n* (%)	5 (9)	123 (21)
Early signs of ischemia on NCCT, *n* (%)	13 (23)	160 (27)
Perfusion deficit present on CTP, *n* (%)	27 (51)	383 (68)
CBV ASPECTS, median (Q1–Q3)	10 (9–10)	10 (7–10)
CBV ASPECTS ≤ 7, *n* (%)	5 (9)	143 (25)
MTT ASPECTS, median (Q1–Q3)	10 (8–10)	8 (4–10)
MTT ASPECTS ≤ 7, *n* (%)	10 (19)	251 (45)
ICA stenosis > 70% on CTA	12 (22)	125 (22)
Occlusion on CTA, *n* (%)	22 (39)	329 (57)
Clot burden score, median (Q1–Q3)	10 (9–10)	10 (7–10)
Poor collateral score, *n* (%)	6 (11)	102 (18)
TOAST classification		
Large artery atherosclerosis, *n* (%)	18 (32)	185 (32)
Cardioembolism, *n* (%)	5 (9)	114 (20)
Small-vessel disease, *n* (%)	11 (20)	54 (9)
Other, *n* (%)	4 (7)	49 (8)
Unknown, *n* (%)	18 (32)	180 (31)

*SD*, standard deviation; *mRS*, modified Rankin scale; *NIHSS*, National Institutes of Health stroke scale; *TIA*, transient ischemic attack; *MI*, myocardial infarction; *PAD*, peripheral artery disease; *NCCT*, non-contrast CT; *CTP*, CT perfusion; *CBV*, cerebral blood volume; *ASPECTS*, Alberta Stroke Program Early CT score; *MTT*, mean transit time; *ICA*, internal carotid artery

### Model building

The main clinical predictors of ischemic stroke recurrence were male sex, history of hyperlipidemia, and history of either stroke or TIA (Table 2). The c-statistic of the clinical model, which contained male sex, history of hyperlipidemia, and history of stroke or transient ischemic attack, was 0.61. Compared with the clinical model, the extended model, which contained previous cerebral infarcts on non-contrast CT and ASPECTS greater than 7 on mean transit time maps derived from CTP, had higher discriminative performance (c-statistic 0.65, *P* = 0.01).

**Table 2:** Effect estimates of the full and simplified clinical models and the extended model for predicting recurrent ischemic stroke

Candidate predictor	Full clinical HR (95% CI)	Simplified HR (95% CI)	Full extended HR (95% CI)	Simplified HR (95% CI)
Age > 65	1.5 (0.8–2.8)	1.3 (0.7–2.3)	1.2 (0.7–2.2)	-
Male sex	1.7 (0.9–3.2)	1.9 (1.1–3.5)	1.7 (0.9–3.2)	1.8 (0.98–3.3)
Hypertension	1.0 (0.5–1.8)	-	-	-
Diabetes	0.9 (0.4–2.0)	-	-	-
Current smoking	0.7 (0.4–1.5)	-	-	-
History of hyperlipidemia	2.0 (1.01–3.9)	1.7 (0.98–3.0)	1.6 (0.9–2.9)	1.8 (1.03–3.2)
History of stroke/TIA	2.1 (1.1–3.9)	2.4 (1.3–4.1)	2.1 (1.1–3.8)	2.0 (1.1–3.6)
History of MI	0.9 (0.4–2.1)	-	-	-
History of AF	0.9 (0.4–2.0)	-	-	-
History of PAD	1.0 (0.3–3.3)	-	-	-
NIHSS ≥ 7	0.7 (0.4–1.4)	-	-	-
Lacunar stroke (TOAST)	0.6 (0.3–1.2)	0.6 (0.3–1.1)	0.6 (0.3–1.3)	-
Previous cerebral infarct on NCCT			1.5 (0.9–2.7)	1.5 (0.9–2.7)
Hyperdense vessel sign on NCCT			0.7 (0.2–2.3)	-
CBV ASPECTS > 7			1.1 (0.3–4.2)	-
MTT ASPECTS > 7			2.8 (0.97–8.0)	2.3 (1.2–4.7)
ICA stenosis > 70% on CTA			1.8 (0.9–3.8)	-
Occlusion on CTA			0.8 (0.4–1.6)	-
Poor collateral score			0.6 (0.2–1.7)	-
C-statistic		0.67		0.70
Optimism-corrected c-statistic		0.61		0.65
Calibration slope		0.70		0.72

*CI*, confidence interval; *TIA*, transient ischemic attack; *NCCT*, non-contrast CT; *CBV*, cerebral blood volume; *ASPECTS*, Alberta Stroke Program Early CT score; *MTT*, mean transit time; *ICA*, internal carotid artery

The NRI for the recurrence group was 0.40 (95% CI − 0.04–0.73) and 0.04 (95% CI − 0.13–0.54) for the non-recurrence group. Taken together, the total NRI was significant (0.44, 95% CI 0.14–0.74). The discrimination slope also improved significantly (IDI 0.03, 95% CI 0.01–0.09).

**Fig. 1:**
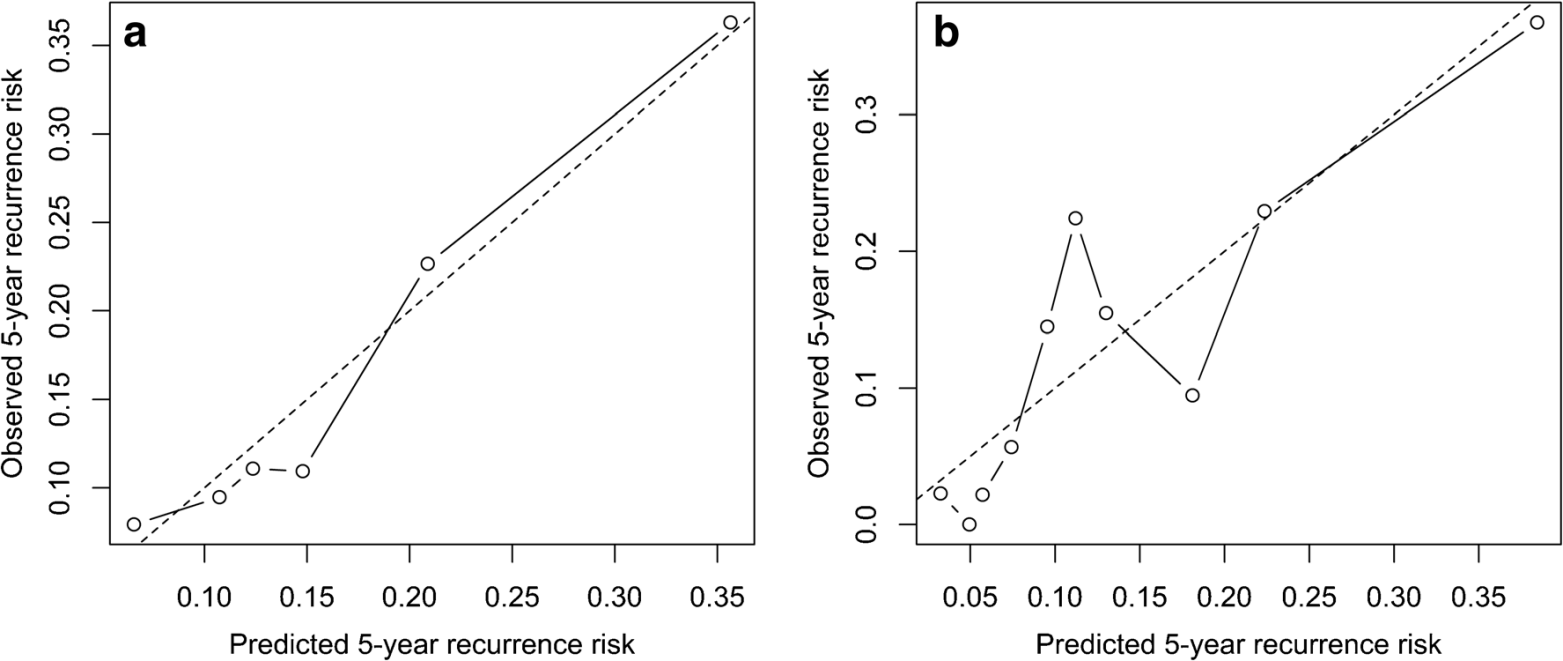
Calibration plot for the clinical model (**a**) and the extended model (**b**) containing clinical and CT-derived predictors. A calibration slope of 1.0 indicates perfect calibration (dotted line)

The calibration plots are shown in Fig. 1. According to the slopes of the clinical (0.70) and the extended (0.72) models, the models were reasonably calibrated.

## Discussion

In this study, addition of two CT imaging variables (previous cerebral infarcts on NCCT and ASPECTS on mean transit time maps derived from CTP) to the clinical model resulted in a significant improvement in discrimination performance for ischemic stroke recurrence over 5-year follow-up.

CT is often performed in patients with ischemic stroke as CT has some advantages over MRI in the acute stroke setting such as the short acquisition time, patient compatibility, costs, and availability. Besides diagnostic purposes, findings on admission CT can be used for prognostic purposes in patients with acute ischemic stroke. For instance, several studies investigated the value of CT findings on predicting clinical outcome after ischemic stroke [13, 25]. The added value of predictors derived from CTP and CTA for predicting clinical outcome after 3 months was limited in the DUST dataset [13]. A previous study showed that prediction of recurrent ischemic stroke at 90 days was significantly improved by adding MRI-derived predictors such as multiple infarcts and involvements of multiple vascular territories to a clinical model [8]. In addition, similar MRI-derived predictors were identified as being predictive of recurrent ischemic stroke during a 2-year follow-up [9]. However, the added value of CT-derived predictors has never been investigated for predicting recurrent ischemic stroke beyond 2 years. In this study, we showed that recurrence risk estimation can be significantly improved by adding CT-derived predictors to a model incorporating clinical predictors.

Although stroke subtype was a significant predictor of recurrent ischemic stroke in previous studies, this predictor did not have added value to the clinical prediction model in our study population [4]. The stroke etiology was determined using the TOAST classification [14]. Determination of the stroke etiology often requires extensive diagnostic work-up and can sometimes only be accurately determined during a follow-up period after the stroke, whereas immediate recurrence prediction after the index event is desirable [26]. In this study, the TOAST classification was determined during the initial admission phase. As follow-up studies for determining the final stroke etiology were not routinely taken into account, the cause of the stroke remained unknown for 31% of the cases. Therefore, the results from this particular analysis need to be interpreted with caution. Future studies should elucidate whether stroke subtype has added value to long-term prediction of recurrent stroke.

The added value of previous cerebral infarcts on NCCT to a history of either stroke or TIA can be explained by the fact that brain ischemia may occur without the patient noticing, which is called a silent brain infarction. The association between silent brain infarction and future stroke has been established before, but it has never been related to recurrent ischemic stroke [27]. Whether the patient has had a previous ischemic stroke or TIA is usually evaluated by history taking. However, it is possible that ischemic brain damage is present, although the patient has not experienced any stroke symptoms. This is a typical example of how CT imaging has added prognostic value to clinical assessments such as history taking.

Intuitively, prediction models will be more reliable if they include predictors that are already well-established risk factors for recurrent stroke. We found that poor collaterals did not seem to be an important predictor for recurrent stroke. This finding does not mean that poor collateral is not a risk factor for recurrent stroke by itself, but, instead, this factor has no added value to the prediction of recurrent stroke beyond the other predictors used in our risk prediction model. An explanation for this finding might be the lack of power due to the small number of patients with poor collaterals. This finding needs verification in a more balanced population.

The observed association between higher ASPECTS on mean transit time maps derived from CTP and increased recurrence risk is surprising, because it implies that patients with smaller areas of ischemia and/or involvement of less ASPECTS regions face a higher risk of recurrence compared with patients with greater areas of ischemia and/or involvement of more ASPECTS regions. We were not able to distinguish between the infarct size and the multiplicity of the infarct as these data were not routinely collected in the DUST. Additional studies are warranted to confirm this remarkable finding and to assess its relation to infarct size, multiplicity, and etiology. An advantage of using ASPECTS is that it can be accurately graded in the acute stroke phase and that it can be instantly used for prediction purposes. Although ASPECTS has been initially developed for NCCT assessments, it can also be applied to other CT modalities such as CTP [15, 28]. In this study, the dichotomized measure of ASPECTS had added value to the clinical prediction model, making it a promising tool for recurrence prediction purposes. This finding however needs verification in a larger study with prospective outcome evaluation.

In this study, we showed that recurrence prediction after ischemic stroke can be improved by using imaging information in addition to clinical information. However, even after the model was improved, the performance was still moderate. Some steps need to be taken before a model that predicts recurrent ischemic stroke can be used in routine stroke care. First, studies may look for additional predictors (e.g., derived from imaging) to see if a clinically relevant improvement can be achieved. For example, current studies are also focusing on including the heart in the stroke admission scan to improve the early diagnosis of cardioembolic causes. Preferably, the found predictors such as ASPECTS on MTT maps should be validated in a separate study cohort. Second, once a model with a sufficiently high performance is developed, it needs to be validated in other cohorts. Third, ideally, the impact of the model needs to be quantified in a randomized controlled trial. In this way, the prognostic model may guide treatment decisions and therefore affect patient outcomes. This study contributes to the process of finding an optimal model for recurrence prediction.

Strengths of this study were the long-term follow-up and the selection of candidate predictors, which were based on literature. In this way, we avoided selecting predictors purely on significant *P* values. In addition, predictor information was collected, prospectively leading to a minimal number of missing values. A limitation of this study was the retrospective collection of follow-up data, which could have induced underestimation of the outcome prevalence. This could have influenced our results in case certain associations are related to the loss of follow-up. For instance, recurrences, which were recorded in another hospital than the hospital of the index stroke, were missed. We do not believe that this has happened often, as most patients return to their own hospital for follow-up visits. Still, the observed prevalence is in line with previous studies, but studies with prospective follow-up are needed to verify our findings. The number of outcomes was relatively small, which was also a limitation of this study. Heart failure was not collected as a potential predictor in this study, whereas it showed to be of predictive value in previous studies [29, 30]. However, with less than sixty recurrences, we were not allowed to add more than five predictors to our extended model. Selecting only three out of fourteen DUST centers contributed to this limitation, but acquiring follow-up data from the other DUST centers was not deemed feasible. Studying larger cohorts may allow more predictors into the final model. Instead of improving a previously developed model, we had to create our own clinical model that best fitted our data. A drawback of this method is that our clinical model needs validation in other studies, whereas a previously developed model has already been validated.

In conclusion, clinical models for predicting long-term recurrence after ischemic stroke have moderate performance and can be improved by adding CT-derived predictors.

## Electronic supplementary material

ESM 1 (PDF 167 kb).

## Data Availability

Descriptive data that support the findings of this study are available from the corresponding author on reasonable request.
