# Supplementary material for: Prediction of long-term recurrent ischemic stroke: the added value of non-contrast CT, CT perfusion, and CT angiography
Source: Neuroradiology. 2020 Aug 28;63(4):483–90. doi: 10.1007/s00234-020-02526-5 (PMC7966192; doi:10.1007/s00234-020-02526-5)
Supplement: Supplementary file 1 — (PDF 167 kb). [file 234_2020_2526_MOESM1_ESM.pdf]

SUPPLEMENTAL MATERIAL

Supplemental Tables

Supplemental Table I. Patient characteristics stratified by included and excluded centers, which participated in the Dutch acute stroke study (DUST)

**Characteristic**	**Included** n=638	**Excluded** n=755
Age, mean±SD	67±14	67±14
Male sex, n (%)	365 (57)	431 (57)
mRS≥3 before stroke	44 (7)	39 (5)
Admission NIHSS, median (Q1-Q3)	6 (3-12)	6 (3-12)
Intravenous thrombolysis, n (%)	390 (61)	479 (63)
Endovascular treatment, n (%)	41 (6)	40 (5)
**Medical history**		
Hypertension, n (%)	319 (50)	408 (54)
Diabetes mellitus, n (%)	84 (13)	129 (17)
Hyperlipidemia, n (%)	190 (30)	267 (35)
Smoking currently, n (%)	193 (32)	188 (25)
Former smoking, n (%)	176 (29)	237 (31)
Never smoked, n (%)	228 (38)	245 (33)
Atrial fibrillation, n (%)	87 (14)	90 (12)
Anticoagulant medication, n (%)	94 (15)	72 (10)
History of stroke/TIA, n (%)	136 (21)	206 (27)
History of MI, n (%)	75 (12)	95 (13)
History of PAD, n (%)	34 (5)	48 (6)
**Imaging findings**		
Old infarcts on NCCT, n (%)	194 (30)	214 (28)
Hyperdense vessel sign, n (%)	128 (20)	155 (21)
Early signs of ischemia on NCCT, n (%)	173 (27)	170 (23)
Perfusion deficit present on CTP, n (%)	410 (66)	387 (51)
CBV (pc)ASPECTS, median (Q1-Q3)	10 (8-10)	10 (8-10)
CBV (pc)ASPECTS ≤7, n (%)	148 (24)	138 (18)
MTT (pc)ASPECTS, median (Q1-Q3)	9 (4-10)	9 (5-10)
MTT (pc)ASPECTS ≤7, n (%)	261 (42)	249 (33)
ICA stenosis >70% or occlusion on CTA	137 (22)	108 (14)
Occlusion on CTA, n (%)	351 (55)	362 (48)
Clot burden score, median (Q1-Q3)	10 (8-10)	10 (8-10)
Poor collateral score, n (%)	108 (17)	64 (9)
**TOAST classification**		
LAA, n (%)	203 (32)	217 (29)
CE, n (%)	119 (19)	116 (15)
SVD, n (%)	65 (10)	118 (16)
Other, n (%)	53 (8)	50 (7)
Unknown, n (%)	198 (31)	254 (34)
**Follow-up**		
Poor clinical outcome at 90 days*, n (%)	254 (40)	247 (33)

* defined as modified Rankin scale equal or greater than 3
DUST indicates Dutch Acute Stroke Study; SD, standard deviation; mRS, modified Rankin Scale; NIHSS, National Institutes of Health Stroke Scale; TIA, transient ischemic attack; MI, myocardial infarction; PAD, peripheral artery disease; NCCT, non-contrast CT; CBV, cerebral blood volume; ASPECTS, Alberta Stroke Program Early CT Score; MTT, mean transit time; ICA, internal carotid artery.
